# Supplementary material for: CLIMB: High-dimensional association detection in large scale genomic data
Source: Nat Commun. 2022 Nov 12;13:6874. doi: 10.1038/s41467-022-34360-z (PMC9653391; doi:10.1038/s41467-022-34360-z)
Supplement: Supplementary file 2 — Description of Additional Supplementary Files [file 41467_2022_34360_MOESM2_ESM.pdf]

Description of Additional Supplementary Files

File Name: Supplementary Data 1
Description: List of differentially expressed genes identified by CLIMB and DESeq2 in each of the studied lineages.

File Name: Supplementary Data 2
Description: Full GO analysis results for differentially expressed genes identified by CLIMB in the erythroid lineage.

File Name: Supplementary Data 3
Description: Full GO analysis results for differentially expressed genes identified by CLIMB in the megakaryocytic lineage.

File Name: Supplementary Data 4
Description: Full GO analysis results for differentially expressed genes identified by CLIMB in the myeloid lineage.

File Name: Supplementary Data 5
Description: Full GO analysis results for differentially expressed genes identified by DESeq2 in the erythroid lineage.

File Name: Supplementary Data 6
Description: Full GO analysis results for differentially expressed genes identified by DESeq2 in the megakaryocytic lineage.

File Name: Supplementary Data 7
Description: Full GO analysis results for differentially expressed genes identified by DESeq2 in the myeloid lineage.

File Name: Supplementary Data 8
Description: Identifiers for subset of DNase-seq experiments used from Meuleman et al. (2020)
