# Supplementary material for: CLIMB: High-dimensional association detection in large scale genomic data
Source: Nat Commun. 2022 Nov 12;13:6874. doi: 10.1038/s41467-022-34360-z (PMC9653391; doi:10.1038/s41467-022-34360-z)
Supplement: Supplementary file 11 — Reporting Summary [file 41467_2022_34360_MOESM11_ESM.pdf]

nature portfolio

Corresponding author(s): Qunhua Li, Ross Hardison

Last updated by author(s): Oct 19, 2022

Reporting Summary

Nature Portfolio wishes to improve the reproducibility of the work that we publish. This form provides structure for consistency and transparency in reporting. For further information on Nature Portfolio policies, see our Editorial Policies and the Editorial Policy Checklist.

Statistics

For all statistical analyses, confirm that the following items are present in the figure legend, table legend, main text, or Methods section.

n/a	Confirmed	
☐	☒	The exact sample size (*n*) for each experimental group/condition, given as a discrete number and unit of measurement
☐	☒	A statement on whether measurements were taken from distinct samples or whether the same sample was measured repeatedly
☐	☒	The statistical test(s) used AND whether they are one- or two-sided<br>*Only common tests should be described solely by name; describe more complex techniques in the Methods section.*
☒	☐	A description of all covariates tested
☐	☒	A description of any assumptions or corrections, such as tests of normality and adjustment for multiple comparisons
☐	☒	A full description of the statistical parameters including central tendency (e.g. means) or other basic estimates (e.g. regression coefficient) AND variation (e.g. standard deviation) or associated estimates of uncertainty (e.g. confidence intervals)
☐	☒	For null hypothesis testing, the test statistic (e.g. *F*, *t*, *r*) with confidence intervals, effect sizes, degrees of freedom and *P* value noted<br>*Give P values as exact values whenever suitable.*
☐	☒	For Bayesian analysis, information on the choice of priors and Markov chain Monte Carlo settings
☒	☐	For hierarchical and complex designs, identification of the appropriate level for tests and full reporting of outcomes
☐	☒	Estimates of effect sizes (e.g. Cohen's *d*, Pearson's *r*), indicating how they were calculated

Our web collection on statistics for biologists contains articles on many of the points above.

Software and code

Policy information about availability of computer code

Data collection	No software was used for data collection.
Data analysis	The following software tools were use to produce the analyses:<br><br>BEDtools v2.29.2<br>Bowtie v0.12.8<br>deepTools v3.5.1<br>DESeq2 v1.36<br>GREAT v4<br>julia v1.5.3<br>LEMON Graph Library v1.3.1<br>MACS v1.3.7.1<br>mashr v0.2<br>NMF v0.23 (R package)<br>PANTHER GO resource v14<br>R v4.1.0<br>RSEM v1.2.28<br>S3norm v1<br>samtools v1.3

SCREEN (no version number)
STAR v2.5.1b_modified
STREME v5.4.1

For manuscripts utilizing custom algorithms or software that are central to the research but not yet described in published literature, software must be made available to editors and reviewers. We strongly encourage code deposition in a community repository (e.g. GitHub). See the Nature Portfolio guidelines for submitting code & software for further information.

Data

Policy information about availability of data

All manuscripts must include a data availability statement. This statement should provide the following information, where applicable:
- Accession codes, unique identifiers, or web links for publicly available datasets
- A description of any restrictions on data availability
- For clinical datasets or third party data, please ensure that the statement adheres to our policy

The data analyzed in this paper are available at NCBI's Gene Expression Omnibus (https://www.ncbi.nlm.nih.gov/geo/) under accession code GSE156074 (https://0-www-ncbi-nlm-nih-gov.brum.beds.ac.uk/geo/query/acc.cgi?acc=GSE156074). This GEO Series includes annotated links to all the CTCF ChIP-seq files, the RNA-seq files, and the DNase-seq files. The DNAase-seq peaks were retrieved from Zenodo (https://doi.org/10.5281/zenodo.3838751). The list of identifiers for the subset of samples analyzed in this paper are in Supplementary File 9.

Human research participants

Policy information about studies involving human research participants and Sex and Gender in Research.

Reporting on sex and gender	N/A
Population characteristics	N/A
Recruitment	N/A
Ethics oversight	N/A

Note that full information on the approval of the study protocol must also be provided in the manuscript.

Field-specific reporting

Please select the one below that is the best fit for your research. If you are not sure, read the appropriate sections before making your selection.

☒ Life sciences ☐ Behavioural & social sciences ☐ Ecological, evolutionary & environmental sciences

For a reference copy of the document with all sections, see nature.com/documents/nr-reporting-summary-flat.pdf

Life sciences study design

All studies must disclose on these points even when the disclosure is negative.

Sample size	No sample size calculation was performed. Analyses were performed on data with 1-3 replicates per condition.
Data exclusions	No data were excluded from datasets used.
Replication	Reproducibility of results were shown by analyzing multiple datasets (e.g., comparing results from 2 different chromosomes).
Randomization	The experimental groups were objectively determined, e.g., examining ChIP-seq data or RNA-seq across multiple cell types at different stages of differentiation. No randomization was used.
Blinding	There was not a specific end-point or read-out to this study, so blinding was not relevant.

Reporting for specific materials, systems and methods

We require information from authors about some types of materials, experimental systems and methods used in many studies. Here, indicate whether each material, system or method listed is relevant to your study. If you are not sure if a list item applies to your research, read the appropriate section before selecting a response.

Materials & experimental systems

n/a	Involved in the study
☒	☐ Antibodies
☒	☐ Eukaryotic cell lines
☒	☐ Palaeontology and archaeology
☒	☐ Animals and other organisms
☒	☐ Clinical data
☒	☐ Dual use research of concern

Methods

n/a	Involved in the study
☐	☒ ChIP-seq
☒	☐ Flow cytometry
☒	☐ MRI-based neuroimaging

ChIP-seq

Data deposition

☒ Confirm that both raw and final processed data have been deposited in a public database such as GEO.

☒ Confirm that you have deposited or provided access to graph files (e.g. BED files) for the called peaks.

Data access links
May remain private before publication.

https://www.ncbi.nlm.nih.gov/geo/query/acc.cgi?acc=GSE156074

Files in database submission

GSE156074_CTCF_classes.bed.gz, GSE156074_RAW.tar, GSE156074_climbCtcfSigCh12.bw, GSE156074_climbCtcfSigEr4.bw, GSE156074_climbCtcfSigEry.bw, GSE156074_climbCtcfSigEryfl.bw, GSE156074_climbCtcfSigHpc7.bw, GSE156074_climbCtcfSigMel.bw, GSE156074_climbCtcfSigMono.bw, GSE156074_climbCtcfSigNeu.bw, GSE156074_climbCtcfSigTcd4.bw, GSE156074_climbCtcfSigTcd8.bw

Genome browser session
(e.g. UCSC)

https://main.genome-browser.bx.psu.edu/cgi-bin/hgTracks?
hgS_doOtherUser=submit&hgS_otherUserName=cak142&hgS_otherUserSessionName=Koch_CLIMB_mm10

Methodology

Replicates

The data used in this manuscript is a re-analysis of already published datasets. Some of the cell types, including LSK (2), CH12 (2), G1E-ER4+E2 (2), erythroblasts (3), MEL (2), and monocytes had replicates available. Only one replicate was available for several rare cell types iMk, CMP, GMP, MEP, CFUMk, and CFUE. Additionally, only one replicate was used for T_CD4, T_CD8, and G1E cells.

Sequencing depth

ID,Target,Cell type,Total reads,Mapped reads mm10,Read length bp,Sequencing parameters
1925,CTCF,G1E,42587210,34060317,50,single end
1299,CTCF,LSK,28398021,27118221,50,single end
1295,CTCF,LSK,82541801,79058840,50,single end
1908,CTCF,iMk,35330050,33568039,50,single end
1976,CTCF,CMP,30986124,28925148,50,single end
1977,CTCF,GMP,29603542,28200655,50,single end
1978,CTCF,MEP,32550070,30632592,50,single end
1979,CTCF,CFUMk,27896499,25821404,50,single end
1980,CTCF,CFUE,26850438,25531371,50,single end
35,CTCF,CH12,14758965,14404662,36,single end
47,CTCF,CH12,24993861,24294384,36,single end
25,CTCF,G1E-ER4+E2,14336356,13957215,36,single end
76,CTCF,G1E-ER4+E2,13857723,12927456,41,single end
100146,CTCF,erythroblasts,77792483,60523200,51,single end
100162,CTCF,erythroblasts,23408126,22098595,50,single end
100163,CTCF,erythroblasts,23637956,22789117,50,single end
44,CTCF,MEL,21649898,20950673,36,single end
69,CTCF,MEL,33289675,31242619,41,single end
100164,CTCF,monocytes,25175692,23464891,36,single end
100166,CTCF,monocytes,31232109,28948689,36,single end
100151,CTCF,neutrophils,16574362,15638199,50,single end
100157,CTCF,T_CD4,38156242,37438700,36,single end
100158,CTCF,T_CD8,7349830,6894634,39,single end
100127,CTCF,HPC7,40085503,37929499,37,single end

Antibodies

Millipore 07-729

Peak calling parameters

Wiggle and peaks called using MACS with parameters --format BAM --gsize 1870000000 --tsize 36 --bw 120 --mfold 12 --wig --space 1
Filter blacklist regions from peaks, and convert the *peaks.xls file from MACs to broadpeak format (see UCSC Genome Browser for format specs)
S3norm version 1(https://github.com/guanjue/S3norm) and default parameters except -r max1

Data quality

ID,Target,Cell type,FRiP score
1299,CTCF,LSK,0.003
1295,CTCF,LSK,0.038
35,CTCF,CH12,0.07
47,CTCF,CH12,0.05
25,CTCF,G1E-ER4+E2,0.13
76,CTCF,G1E-ER4+E2,0.18
100146,CTCF,erythroblasts,0.28
100162,CTCF,erythroblasts,0.41
100163,CTCF,erythroblasts,0.33
44,CTCF,MEL,0.110
69,CTCF,MEL,0.144
100164,CTCF,monocytes,0.09
100166,CTCF,monocytes,0.12
1925,CTCF,G1E,0.352
1908,CTCF,iMk,0.054
1976,CTCF,CMP,0.097
1977,CTCF,GMP,0.19
1978,CTCF,MEP,0.29
1979,CTCF,CFUMk,0.17
1980,CTCF,CFUE,0.031
100151,CTCF,neutrophils,0.087
100157,CTCF,T_CD4,0.55
100158,CTCF,T_CD8,0.55
100127,CTCF,HPC7,0.7

Software

Basecalls using bcl2fastq-1.8.4, and parameters --no-eamss --mismatches 1
Mapping to reference genome mm10 canon with Bowtie 1.0.0 using parameters --chunkmbs 1024 -y -n 2 --best -k 1 --maxbts 800 -l 28 -e 80 --sam-nohead --sam
Wiggle and peaks called using MACS with parameters --format BAM --gsize 1870000000 --tsize 36 --bw 120 --mfold 12 --wig --space 1
Filter blacklist regions from peaks, and convert the *peaks.xls file from MACs to broadpeak format (see UCSC Genome Browser for format specs)
S3norm version 1(https://github.com/guanjue/S3norm) and default parameters except -r max1
CLIMB 1.0.0 (https://doi.org/10.5281/zenodo.7121446) with dependent Julia package cgibbs.jl version 1.0.0 (https://doi.org/10.5281/zenodo.7121450). CLIMB was run at the default settings in all analyses.
